# Photoactive Gel for Assisted Cleaning during Olive Mill Wastewater Membrane Microfiltration

**DOI:** 10.3390/membranes7040066

**Published:** 2017-11-25

**Authors:** Yilong Han, Lidietta Giorno, Annarosa Gugliuzza

**Affiliations:** 1 Research Institute of Membrane Technology-National Research Council (ITM-CNR), 87036 Rende, CS, Italy; yilong22@gmail.com (Y.H.); l.giorno@itm.cnr.it (L.G.); 2 Department of Pharmacy, University of Calabria (UNICAL), 87036 Rende, CS, Italy

**Keywords:** self-cleaning, titanium oxide nanoparticles, layer-by-layer, water treatment, submerged microfiltration

## Abstract

A photoactive gel has been fabricated on the surface of polyethylene membranes for enhancing the fouling resistance during olive mill wastewater treatment. Light and pH responsive materials have been introduced in the membrane surface through the build up of a layer-by-layer pattern, which is formed by photocatalytic nanoparticles and ionic polyelectrolytes. The best working conditions to contrast foulants adsorption have been explored and identified. Repulsive interfacial forces and assisted transfer of foulants to catalytic sites have been envisaged as crucial factors for contrasting the decline of the flux during microfiltration. Tests in submerged configuration have been implemented for six continuous hours under irradiation at two different pH conditions. As a result, a worthy efficiency of the photoactive gel has been reached when suitable chemical microenvironments have been generated along the shell side of the membranes. No additional chemical reagents or expensive back-flushing procedures have been necessary to further clean the membranes; rather, fast and reversible pH switches have been enough to remove residues, thereby preserving the integrity of the layer-by-layer (LBL) complex onto the membrane surface.

## 1. Introduction

The olive oil keeps unrivalled nutritional properties and health benefits, since its high content of monounsaturated fatty acids is concerned to strongly reduce the risk of heart disease, high blood pressure, and stroke [1]. However, the squeezing of olives fruits yields every year about 30 million m^3^ of olive mill wastewater (OMW), reach in acids, pectinases, and phenols [2]. The lack of economically competitive and efficient method to manage such an amount of wastewater is expected to negatively impact on the ecosystem due to a severe production of phytotoxic sewage [3]. Within the frame of implementation of eco-sustainable strategies, integrated membrane processes play a key role in the recovery of water with high degree of purity and bioactive compounds [4,5]. Micro/ultrafiltration (MF/UF), nanofiltration (NF), and reverse osmosis (RO) well meet the requirement of eco-friendly and inexpensive approaches for water recovery [6,7]. In the logic of integrated membrane systems, MF represents a first important clarification step for an effective low-pressure filtration of suspended contaminants such as sediments, colloidal particles, macromolecular agglomeration, and by-products of microorganism growth. However, the prolonged exposition of membranes to organic and inorganic compounds brings about inevitably with time pore blocking and/or formation of a dense cake layer onto the membrane surface, resulting in a severe fouling [7,8]. The foremost consequence is a heavy decline of the water flux with a loss of the membrane performance. Generally, the adhesion of colloids and dissolved organic substances takes place rapidly, reaching a steady-state condition, which can degenerate in an irreversible fouling. To contrast this event, expensive practices, such as back-flushing cycles or harsh chemical cleaning treatments, are usually implemented [9,10], providing with time irreversible damages for membranes and additional risks for environment [11].

New solutions concerning novel membrane-types are today requested to improve the process efficiency without impacting on the environment, while time and operational costs may be better managed [12,13,14,15,16]. Nanomaterial-based membranes for water purification are envisaged to be powerful and practical routes for developing high-performing separation processes [17,18,19].

Within the frame of this work, patterned membrane surfaces with dual responsive behavior have been proposed to endorse in-situ cleaning action through gel-assisted photodegradation mechanisms. The interplay of complementary functions has been addressed at yielding chemical microenvironments that are suitable for controlled transfer of foulants to photocatalytic degradation sites through delayed hole-electron pair recombination. As a result, foulants adhesion has been reduced and the decline of the flux has been successfully contained when favorable combinations of repulsive forces and photodegradation have been reached. 

Precisely, titanium oxide (TiO_2_) nanoparticles (NPs) have been dispersed in polyacrylic acid (PAA) and patterned with poly (diallyldimethyl ammonium chloride) (PDDA) in a hybrid complex that is deposited onto the shell side of polyethylene (PE) hollow-fiber membranes via layer-by-layer (LBL) approach [20,21,22]. Commercial PE membranes, which are considered tricky to manage wastewater due to the very high susceptibility to fouling, have been chosen with the intent to demonstrate the real efficiency of the photoactive gel under specific working conditions. Suitable microenvironments have been generated along the shell side of this kind of membrane in order to examine the photoactive gel aptitude for contrasting large adsorptions of foulants without supplementary cleaning practices. 

Therefore, the responsiveness of the LBL complex has been discerned under different operative conditions. Experiments have been carried out in batch and in continuous by using triggers, such as pH and UV light. Tests of submerged microfiltration have been implemented and a comparison with pristine PE membranes has been done. Every experiment has been run for six hours without stoppages for supplementary chemical cleaning or back-flushing actions. Despite that prolonged contact with foulants solutions, it has been not necessary to use harsh cleaning procedures at the end of each filtration process. A simple switch of pH has been enough to generate repulsive charge throughout the overall surface for removing over-accumulated foulants from the PE-LBL membrane surfaces.

Prospectively, this approach appears to be advantageous, because photocatalytic TiO_2_ NPs that are immobilized on membrane surfaces solve the critical difficulty to separate NPs from aqueous solutions after degradation of contaminants, taking the great chance to combine cleaning and separation steps. Further, the dispersion of TiO_2_ NPs in pH responsive hydrogels yields a larger photoactive surface area, as well as changes in the NPs properties due to gel assistance. As a result, the enhancement of the redox potential can be observed and durable membrane filtration can be accomplished under not usual circumstances as well. 

## 2. Experimental

### 2.1. Materials

Hydrophilic polyethylene (PE) hollow fibers with pore size of 0.4 μm and internal/external diameters of 0.41/0.65 mm have been used to build up submerged configured MF modules (Econity, Yangji-myeon, South Korea). Titanium oxide nanoparticles (99%, Sigma Aldrich Srl, Milan, Italy) together with polyacrylic acid (PAA) (average *M*_w_ = 250,000, Sigma Aldrich Srl, Milan, Italy) and poly(diallyldimethylammonium chloride) (PDDA) (average *M*_w_ = 400,000–500,000, Sigma Aldrich Srl, Milan, Italy) solutions of 10^−2^ M (based on repeat unit molecular weight) have been used to functionalize the shell side of each single hollow fiber according to the LBL method [21,22]. The nanoparticles have been dispersed in PAA solutions under ultrasound treatment for 24 h at a concentration of 0.5 mg mL^−1^, while the pH of each solution has been adjusted to 6.75 by sodium hydroxide. The membranes have been dipped in each solution for 15 cycles deposition. Ultra-pure water (filtered by USF ELGA plant), Glycerol (≥99%, Sigma Aldrich Srl, Milan, Italy) and Diiodomethane (99%, Sigma Aldrich Srl, Milan, Italy) have been used to measure contact angle values and related surface free tension components. Olive mill wastewater (OMW) has been supplied by the “Olearia San Giorgio” industry, located in Calabria and used after centrifugation at 6000 RMP for 45 min (South of Italy).

### 2.2. Methods

The membrane morphology has been investigated by scanning electron microscopy (SEM; Quanta 200, FEI Company, Oberkochen, Germany), while attenuated total reflectance (ATR) spectra have been directly collected from the membrane surfaces over different points of the sampling area and have been recorded at a resolution of 4 cm^−1^ (UATR crystal Diamond/ZnSe—Spectrum One System Perkin-Elmer Instruments, Hillsboro, OR, USA) in order to get an indication about functionalization and chemical stability, as well as adsorption and self-cleaning efficiency of the LBL complex under different experimental conditions. The membrane wettability has been assessed through static contact angle measurements by using a CAM200 according to the capillary method (KSV instrument LTD, Helsinki, Finland), while the membrane affinity, expressed as a difference of parameters of solubility (Δ*δ*), has been analyzed from energy cohesive and related surface free energies according to Good and van Oss approach [23,24]. In detail, the Lifshitz-van der Waals *γ^LW^* [mJ/m^2^], polar *γ^AB^* [mJ/m^2^], acid (electron acceptor) *γ*^+^ [mJ/m^2^], base (electron donor) *γ*^−^ [mJ/m^2^] components of the surface free energy *γ* [mJ/m^2^] have been calculated from mean values of the contact angles according to the following equations:

(1) $$\gamma_{s}^{LW}=\frac{\gamma_{l}^{LW}\cdot {\left(1+cos\theta \right)}^{2}}{4}$$

(2) $$\gamma_{l}\cdot \left(1+cos\theta \right)=2\cdot \left(\sqrt{\gamma_{s}^{LW}\cdot \gamma_{l}^{LW}}+\sqrt{\gamma_{s}^{+}\cdot \gamma_{l}^{-}}+\sqrt{\gamma_{s}^{-}\cdot \gamma_{l}^{+}}\right)$$

(3) $$\gamma_{s}^{AB}=2\cdot \sqrt{\gamma_{s}^{+}\cdot \gamma_{s}^{-}}$$

where *s* and *l* are referred to solid and liquid, respectively. The results have been the averages of 8–10 different measurements for each kind of solvent. The energy cohesion density (*e_coh_*, 10^6^ J/m^3^) has been calculated from the overall surface free tension, while the solubility parameters (*δ*, 10^3^ J^1/2^/m^3/2^) have been estimated from the energy cohesion density, according to the following relations:

(4) $$\gamma_{s}=0.75\cdot {\left(e_{coh}\right)}^{2/3}$$

(5) $$\delta ={\left(e_{coh}\right)}^{1/2}$$

The pendant drop method has been used to estimate the surface free tension of each single OMW solution. The LBL assembly stability has been tested under magnetically stirring at different pH conditions. For each kind of sample, four tests have been carried out.

The viscosity of OMW solutions (*μ*) has been measured by using a programmable rheometer (Brookfield DV III ULTRA).

Photocatalytic tests have been performed in UV*λ* range of 200–400 nm by using A 250 W high-pressure Hg lamp, placed approximately at 20 cm from the membrane surfaces. The solutions have been continually stirred in the dark and light conditions to establish the adsorption–desorption equilibrium. 

Submerged configured MF experiments have been implemented by using hollow fiber modules without and with a dual responsive LBL complex. For each single module, a number of four PE hollow fiber membranes long 10 cm, and with an overall surface area of 8.2 × 10^−4^ m^2^ have been assembled and completely immersed in the OMW feed solutions. A vacuum pump has been connected with the side glued of the module in order to generate an absolute trans-membrane pressure from shell to lumen within the range of 10 to 60 kPa at 20 °C. The permeation experiments have been run for six continuous hours under irradiation at pH 2.2 and 4.6, respectively.

Average fluxes (*J*) in Lm^−2^h^−1^ have been measured with time, while the relative flux has been calculated as a ratio between the water flux *J* after OMW adsorption and the pure water flux *J_w_* before OMW adsorption, according to the relationship:

(6) $$Relative\;flux=\frac{J}{J_{w}}$$

The membrane resistance (*R*, m^−1^) has been calculated according to the following equation:

(7) $$R=\left(\frac{\Delta P}{\mu \cdot J}\right)$$

where is Δ*P* (Pa) is the transmembrane pressure, *μ* in Pa s the dynamic viscosity of the feed, and *J* in m s^−1^ the membrane flux.

Finally, the decline of the flux (FD) has been calculated according to Equation (8):

(8) $$FD=\left(1-\frac{J_{t_{2}}}{J_{t_{1}}}\right)$$

where *J_t_* is the flux measured at successive running hours.

## 3. Results and Discussion

### 3.1. Membrane Functionalization and Stabilization

An electrostatic layer-by-layer assembly has been chosen to deposit hybrid responsive materials onto the porous surface of commercial PE hollow fibers. A colloidal dispersion of TiO_2_ nanoparticles in PAA aqueous solutions has been prepared at pH 6.75 without adding salts and has been successively patterned with a counter solution of PDDA. During the LBL built up the ionic charges that are distributed along the segment polymer chains of the ionic polyelectrolytes pair has been shielded. 

**Figure 1:**
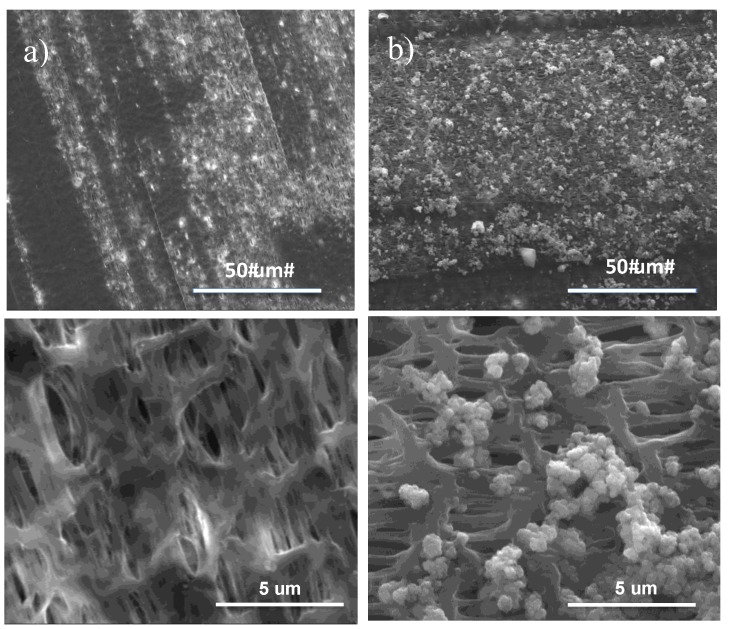
SEM micrographs collected onto the shell side of polyethylene (PE) hollow fiber membranes before (**a**) and after (**b**) (PAA/TiO_2_)_15_(PDDA)_15_ layer-by-layer (LBL) deposition.

SEM micrographs reveal the formation of uniform particulate-like structure with size of 1 to 2 μm, thus leaving open pores over the PE shell side (Figure 1).

**Figure 2:**
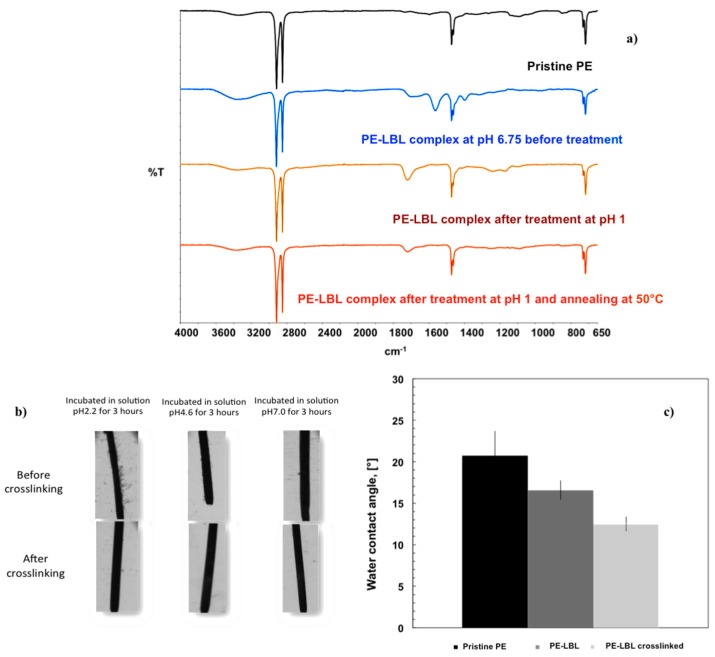
Comparison of attenuated total reflectance (ATR) spectra collected onto PE-LBL membranes before and after cross-linking (**a**); optical pictures related to LBL complex incubated in different buffer solutions (at pH 2.2 to 7.2) before and after cross-linking (**b**); changes in wettability with LBL construction and stabilization (**c**).

Infrared analyses confirm the occurrence of chemical changes through the surface due to the formation of the LBL complex (Figure 2a). From ATR spectra, it is discernable a small broad stretching of carboxylic C=O group, while the appearance of two strong bands at 1563 and 1399 cm^−1^ clearly indicate the presence of a large number of carboxylate anion groups of PAA onto the membrane surface. These two last vibrational modes are associated to asymmetrical and symmetrical stretching bands of the carboxylate anion, respectively.

At pH 6.75, the polyacid reaches an almost full-ionized state, thus causing the polymer chains to elongate in a flat conformation due to the high density of repulsive negative charge along the segments [20]. The largest availability of the carboxylate anion enables PAA to shield the oppositely charged sub-layer (PDDA) leading to a major saturation of the oppositely charged sites with a reduced amount of material. Accordingly, PE pore occlusion has been successfully prevented. 

The chemical stability of the LBL complex has been also examined within a broad range of pH; a certain tendency to leach has been observed with time, especially at the lowest pH value. Initially, the LBL is in a swelled state and the access of additional water molecules into the bulk is facilitated, thus yielding the polymer assembly more exposed to competitive intermolecular interactions that can affect the stability of the assembly locally. With this concern, the LBL complex has been, therefore, dipped in a buffer solution at pH 1.0 for a few seconds in order to promote cross-linking via hydrogen bonding; successively, the membranes have been annealed at 50°C for 3 h and the excess of adsorbed water has been removed so that compactness and chemical stability of LBL assembly have been achieved through strengthened H-bonding and electrostatic interactions between PDDA and PAA (Figure 2). 

The chemical cross-link has been confirmed by the disappearance of two bands at 1563 and 1399 cm^−1^ (νCOO^−^) and the concomitant appearance of the infrared mode associated to the COOH group at ν1720 cm^−1^ (Figure 2a). Optical microscopic observations have confirmed further the stability of LBL complex after 3 h of incubation in buffer solutions at various pH values (Figure 2b).

**Figure 3:**
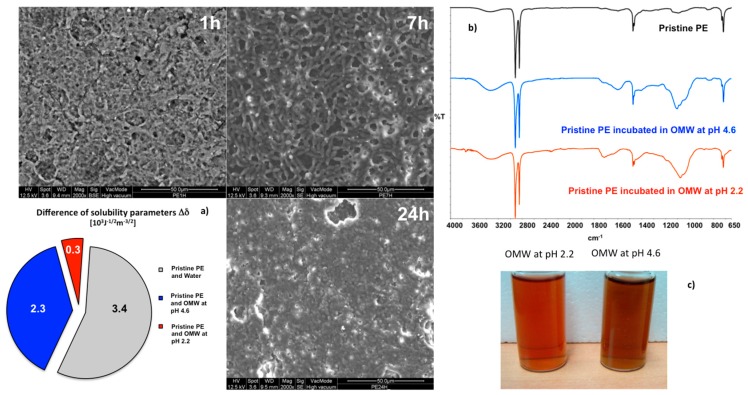
SEM micrograph collected onto the fouled PE membrane surface after 1, 7, and 24 h of incubation in olive mill wastewater (OMW) solution at pH 2.2 and related affinity with various solutions (**a**); ATR spectra collected onto PE membranes incubated in OMW_2.2_ and OMW_4.6_ (**b**); Picture related to OMW solutions at pH 2.2 and 4.6 (**c**).

At this stage, typical signature markers of TiO_2_, that is associated to the stretching vibration of Ti-O-Ti and falling in the fingerprint region of 900–600 cm^−1^, have been not easily discernable from ATR spectra due to the overlapping of two strong rocking vibrations of PE chains (ρCH_2_) at 730 and 718 cm^−1^, respectively. However, the broadness of the bands centered at 3364 and 1717 cm^−1^ together with the appearance of a shoulder around 1409 cm^−1^ suggest the occurrence of different molecular interactions due to the establishment of heterogeneous chemical environments that receive also the contribution of O–H bond of the surface-adsorbed species of particles (Ti–OH) [25], plausibly. It is further interesting to observe that the cross-linked LBL surfaces confer hydrophilic character to the membranes due to a higher availability of hydrogen acceptor/donor groups. A reduction of 8° for contact angle values can be appreciated moving from pristine to cross-linked PE-LBL membranes (Figure 3b). 

### 3.2. Experiments in Batch

Raw OMW solutions deriving from the squeeze of olive fruits are somewhat complex mixtures. They contain acids, pectinase, and phenols, but also suspended solids and particles. So, pretreatments have been necessary before processing. The solutions have been filtered with a metal mesh having a cut off of 35 μm in order to remove larger suspended particles. The filtrate has been centrifuged at 6000 rmp for 45 min and the suspension has been examined by UV-Vis in order to estimate the content and composition in phenols: 2678 mg L^−1^ against 2727 mg L^−1^ valued for no-centrifuged solutions. The original OMW solution, which looks like turbid, exhibits a pH of 4.6 and a dynamic viscosity value of 0.00181 Pas. After adjusting the pH value to 2.2, a further clarification of the solution reduces the dynamic viscosity to 0.00168 Pas (Figure 3c).

As reported in a recent study [26], the further acidification causes a destabilization of the suspension by modifying the zeta potential with subsequent easier flocculation of suspended solids. 

Nevertheless, both the OMW solutions have been used to explore the foulants adsorption on pristine PE and PE-LBL membrane surfaces in order to detect the role of the density of charge in the control of interfacial phenomena. Accordingly, pristine PE membranes have been dipped for 1, 7, and 24 h under stirring in both the OMW solutions at two different pH values. 

SEM images collected onto the surfaces reveal the formation of a film, which grows with time occluding increasingly the pores of the membrane. This large susceptibility to the foulants adsorption can be regarded as the result of an extensive affinity between membrane and solutions (Figure 3a). At the lowest pH conditions, the smallest difference can be estimated between the parameters of solubility (Δ*δ*) of PE membranes and OMWs. As explained hereafter, this parameter is analyzed through the calculation of overall surface free tensions and related parameters of solubility [23], and is a good indicator of the power of interaction between interfaced systems. ATR spectra confirm further the occurrence of significant interactions between two systems (Figure 3b). Stretching infrared modes falling in the regions of 3000–3500 cm^−1^, 1550–1750 cm^−1^, and 1000–1230 cm^−1^, ascribable to νO-H, νC=O(OH), νC=O(O^−^), and νC(C=O)C groups of species dissolved in OMWs can be easily detected. At pH 4.6, the νC=O bands is significantly shifted towards 1600–1550 cm^−1^ vibrational region, suggesting a higher density of negative charge over the surface due to the increased number of carboxylate anions.

**Figure 4:**
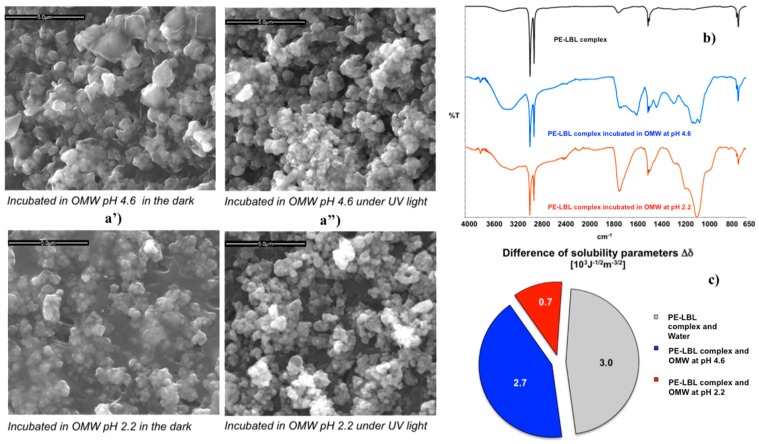
SEM micrographs captured after incubation in the dark (**a’**) and under UV light irradiation (**a”**) the bar is 5.0 μm; ATR spectra collected onto PE-LBL membranes after incubation in the dark (**b**); comparison of the difference between the solubility parameters of PE-LBL membrane and water, OMW_2.2_ and OMW_4.6_ (**c**).

After functionalization, the nanostructured membranes have been again dipped in the dark at two different pH values under continuous stirring in order to reach sorption-desorption equilibrium. SEM micrographs (Figure 4a’), collected onto the PE-LBL membrane surfaces, have showed a discernable formation of a dense film on the surface treated for 24 h at pH 2.2. Although marginally reduced, this adsorption of pollutant continues to be detectable on the surface dipped at pH 4.6 as well. 

Once more, the degree of affinity of the PE-LBL complex to OMW solutions and pure water has been estimated. Figure 4c shows the difference between the parameters of solubility of the membrane surface and water, as well as between the membrane and OMW_2.2_ and OMW_4.6_ solutions, respectively. Provided that this difference is regarded as a quantitative indicator of attraction between two systems coming in contact, small differences mean high affinity, while large differences indicate poor interactions between two systems. The lowest difference value (0.7 × 10^3^ J^−1/2^m^−3/2^) of the solubility parameters can be estimated for the PE-LBL/OMW_2.2_ pair, confirming the strongest interaction between the PE-LBL surface and acidified pollutant solution. Indeed, a strong accumulation of foulants has been detectable on the membrane surface by SEM and infrared analyses (Figure 4a,b). At pH 2.2, a strong and broad vibrational absorption centered at 1708 cm^−1^ (νC=O) and associated with carboxyl acid groups is visible, while a very broad absorption attributed to infrared modes νC=O(O-), νC-C(=O)-O, and νO-C-C can be observed in the region 900–1250 cm^−1^. 

At pH 4.6, a certain affinity continues to be observable, and, even if less intense, broader infrared absorptions (1760–1500, 1263, and 1100 cm^−1^) are still noticed. It is relevant to observe that at a higher pH value (4.6), there is a major density of negative charge in OMW solution but also along the polyelectrolyte PAA segment chains forming the LBL complex. The increase in the density of negative charge is expected to cause higher repulsion between ionic species with similar charge, thus contrasting the formation of heavy foulants adsorption over the membrane surface, differently from what was observed at lower pH conditions (Figure 4a). However, the contrast to the foulants adhesion seems to be not yet satisfactory.

### 3.3. Experiments in Batch under Irradiation

**Figure 5:**
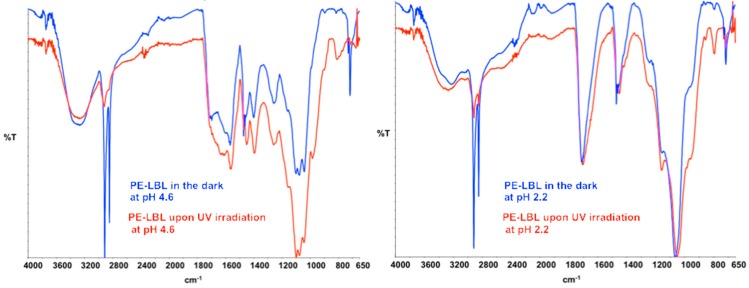
ATR spectra collected onto PE-LBL membranes after incubation in the dark and upon UV light irradiation.

Samples of PE-LBL membranes have been also incubated in OMW solutions for 24 h under continuous irradiation and stirring. After dipping, SEM analysis reveal significantly reduced adsorption of pollutants onto both of the membrane surfaces dipped at pH 2.2 and 4.6 (Figure 4a”), while ATR spectra show interesting variations in the absorption infrared bands (Figure 5). In both of the cases, the irradiation causes broadness, together with the appearance of additional shoulders in the identified infrared bands νC-OH, νC=O, νC=O(OH), νC=O(O-), νC-C(=O)-O, and νO-C-C. Instead, aliphatic stretching modes νCH_2_, νCH_3_ (2917, 2849 cm^−1^), as well as aliphatic bending and rocking modes δCH_2_, δCH_3_, ρCH_2_ (1473, 1463, 1456, 730, 718 cm^−1^) of the segment chains appear to be somewhat reduced in intensity and resolution, while a distinctive broad band looks around 800 cm^−1^.

Concerning samples that are dipped at pH 4.6, the band centered at 1396 cm^−1^ and attributed to symmetrical stretching of carboxylate anion appears to be much more intense, broader, and shifted to lower wavenumbers (Δν, 5 cm^−1^). It should be noted that this wavenumber is also assigned to the O-H bond vibration of the surface-adsorbed species of NPs (Ti–OH) [25]. This important shift suggests, hence, changes in the chemistry of the surface when irradiated. Also, the bands that are assigned to νO-H, νC=O, νCOO,^−^ and νC-O bonds, which are able to coordinate O-H groups of activated TiO_2_ NPs, are shifted towards lower wavenumbers, thus providing a clear indication about light activation. Interestingly, the strong intensification of the band at 800 cm^−1^ could be ascribed to a distortion due to coordination between TiO_2_ NPs and aromatic pollutants. 

In this context, it would be reminded that once excitation occurs across the band gap of NPs, an electron-hole pair is generated, while a charge transfer takes place toward adsorbed species on the NPs surface from solution [27]. The electron is predictable to reduce an electron acceptor, such as dissolved oxygen, whereas the hole migrates to the surface, oxidizing donor species such as hydroxyl species. As a result, superoxide anion (O_2_^•**−**^) and hydroxyl (OH^•^) radicals are generated and photo-degradation takes place reducing the foulants adsorption. Comparing the results achieved in batch conditions, enhanced resistance to contamination can be appreciated at higher pH values because favorable environmental conditions would be formed.

### 3.4. Water Flux Testing

**Figure 6:**
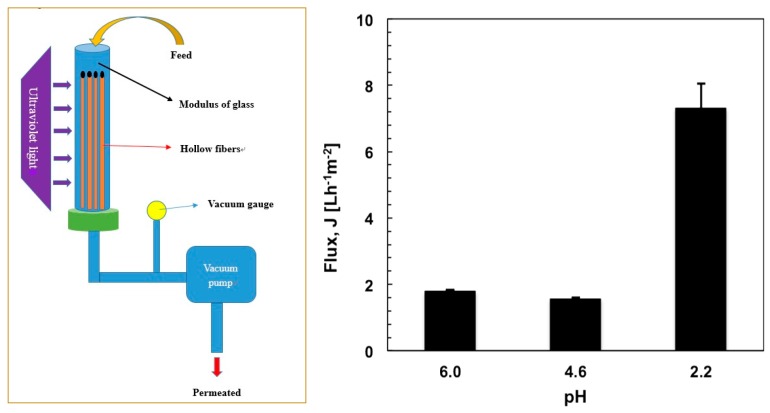
Modulus assembly in submerged configuration microfiltration (**a**) and average pure water flux measured through PE-LBL membranes at different pH values (**b**).

PE-LBL membranes have been assembled in tubular modules and processed in a submerged microfiltration reactor (Figure 6). This takes advantage over a traditional cross-flow microfiltration reactor of reduced power consumption and limited foulants deposition onto the membrane due to low operative pressures [28,29], even if lower fluxes are unfortunately obtained. One side of PE-LBL membranes has been sealed with glue, while the other side has been fixed with glue but left free without further set, as shown in Figure 6a. The shell side of the membranes has been put in direct contact with OMW solution (feed side), while a slight vacuum has been applied to the free side of the fibers in order to cause a lower pressure at the lumen side of the membrane where the permeate has been collected. The absolute trans-membrane pressure (ΔP) has been monitored by a vacuum gauge kept between modulus and vacuum pump.

Figure 6b shows a comparison of pure water flux measured through PE-LBL membranes at 50 kPa under different pH conditions. Higher water flux has been measured at pH 2.2 due to the fact that the LBL complex is in a shrunk state. Because the polymer chains have been interlinked via hydrogen bonding interactions, the volumetric space in proximity to the PE pores is reduced and the resistance to the transport is limited further.

On the contrary, lower fluxes have been estimated at higher pH values due to a higher degree of ionization, which yields increased density of the negative charge. This brings about the polymer chains to expand and swell due to repulsive interactions; as a consequence, additional resistance to the transport is observed. 

Before every experiment, the membranes modules have been, hence, compacted with a buffer at pH 2.2 within an absolute pressure range of 10 to 60 kPa at 20 °C in order to keep chemical, mechanical, and structural stability of the membranes during operation. Three running cycles have been necessary until constant pure water flux (7.1 ± 0.7 Lm^−2^h^−1^) has been reached.

### 3.5. Controlled Fouling during Submerged Microfiltration

Based on experiments carried out in-batch, submerged microfiltration testing has been run under different pH conditions, and UV light irradiation. The membranes have been worked for six hours. No middle cleaning steps or flux of air bubbles have been supplied during the tests with the precise intent to explore the efficiency of the photoactive gel without supplementary assistance.

**Figure 7:**
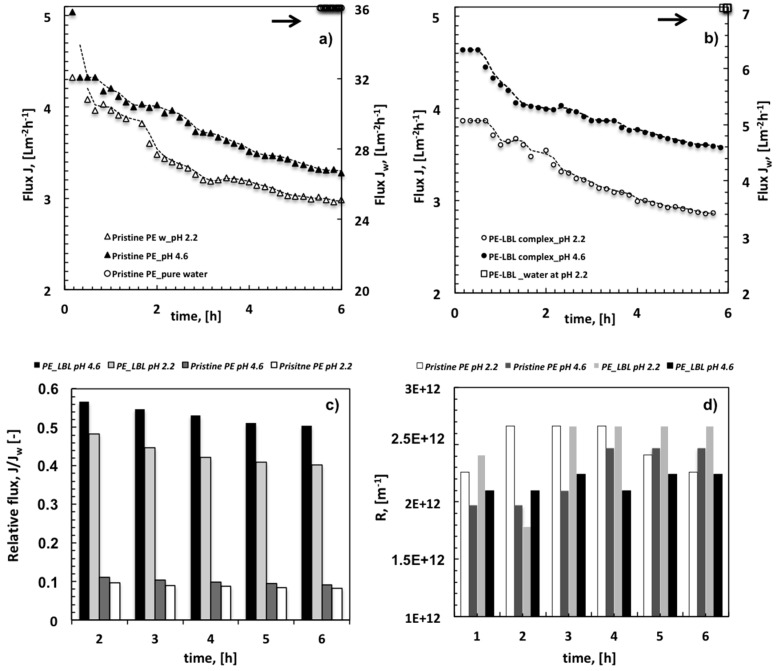
Average Flux (**a**,**b**), relative flux (**c**) and resistance to the flux (**d**) measured with time through pristine PE and PE-LBL membranes coming in contact with pure water and OMW solutions at pH 2.2 and 4.6 and under irradiation.

Figure 7a,b show the average flux measured through pristine PE and PE-LBL membranes coming in contact with water and OMW streams, respectively.

It is relevant to note that the LBL complex causes an expected reduction of the pure water flux due to additional resistance that is induced by the gel deposited on the shell side of the membranes. 

When comparing the performance of each single membrane, it is however observed that the flux falls heavily from 36 ± 3 Lm^−2^h^−1^ (*J_w_*, pure water) to 3.5–3.0 ± 0.2 Lm^−2^h^−1^ (*J*, OMW_4.6–2.2_) for pristine PE membranes, resulting in a relative flux of 0.11 to 0.09 after two first running hours, respectively (Figure 7c). Functionalized membranes show almost halved flux, yielding after 2 h a relative flux of 0.57 at pH 4.6 and 0.48 at pH 2.2.

When considering that a larger affinity to OMW solutions is observed for pristine membranes (Figure 3a), it is also pertinent to examine the changes in the resistance to the flux for each type of membrane (Figure 7d). The latter are, in fact, good indicators of the capability of the membrane to contrast foulants adsorption. 

At pH 2.2, both the pristine and functionalized PE membranes exhibit the highest resistance values. A certain propensity of the LBL complex to contrast foulants adsorption is just perceivable within the first two running hours. Given that the density of the negative charge of the LBL complex is almost negligible at pH 2.2, this initial hard effort to resist fouling could plausibly be ascribed to photodegradation activity, which tends to extinguish with time quickly. 

At pH 4.6, the PE-LBL complex shows a slightly higher resistance value in the first part of the process, due to its tendency to swell. As expected, an inevitable slowing down on the fluid permeation is observed during the first hours of the experiments.

Conversely, after 3 h the increase in the resistance is quite contained and almost constant for LBL complex, whereas it becomes much more marked for PE pristine membranes (Figure 7d). This reversal of behavior can be regarded as an undeniable indicator of the capability of the LBL complex to contrast the decline of the flux with time. 

**Figure 8:**
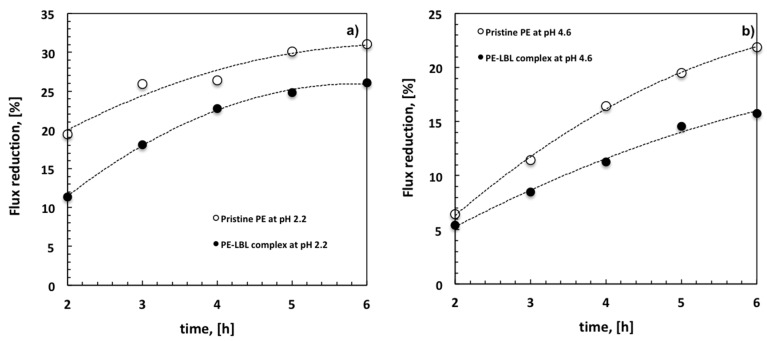
Comparative flux reduction for pristine PE and PE-LBL membranes at pH 2.2 (**a**) and 4.6 (**b**) under irradiation.

With this regard, it is also interesting to analyze the decline of the average flux (FD) with time for all of the membranes under different pH conditions (Figure 8). At pH 2.2, a FD of 19% is detectable for pristine PE membranes against the 11% for LBL membranes after two hours. The decline reaches the 31% for unmodified membranes and a 26% for the modified ones after six hours (Figure 8a). 

Otherwise, when the module is irradiated at pH 4.6, the difference between pristine PE and PE-LBL membranes becomes much more discernable throughout the overall experiment. After six hours, the average flux looks to reduce with time more quickly for pristine PE (22%), while resulting in a slower decrease for PE-LBL membranes (15%) (Figure 8b). This is in full agreement with the resistance behavior. 

This datum becomes much more significant if compared to the decline of the average flux (63%) that is observed after 2 h for microfiltration of OMW with a hydrophilic cellulose acetate membrane (pore size: 0.2 μm, TMP, 18 kPa, T = 25 °C, pH= 5.02) [30].

In full accordance with the experiments carried out in batch, the photocatalysis becomes effective when repulsive interfacial forces are well established between solution and membrane surface, according to the concept of photoactive gel assisted cleaning [31].

At pH 4.6, the density of negative charge causes a partial elongation of the polymer chains; TiO_2_ NPs are pulled out while electrostatic repulsive interactions contrast excessive adsorption of foulants onto the surface. Also, the polyelectrolytes act as dispersing agents and electrostatic stabilizers for TiO_2_, preventing agglomeration and making a larger specific surface area. Further, they assist the transfer to NPs surface, wherein undesired species are more quickly degraded due to the formation of peroxide anion and hydroxyl radicals [32,33,34]. This cooperative mechanism is expected to contrast the accumulation of foulants onto the membrane surface more efficiently, also preserving the action of the photocatalyst with time. 

At pH 2.2, a significant loss of photodegradation efficiency is instead observed with time due to a larger accumulation of foulants onto the surface. Generally, it is found that the photocatalytic activity decreases with increasing reactant concentration, because the active sizes on the catalyst surface are limited [35,36]. Under these conditions, the path length of the photon through a thicker layer of pollutants is decreased, thereby causing quicker hole-electron pair recombination and reduced formation of the charge carrier [37]. The result is a progressive decrease in photodegradation efficiency until a full deactivation of the photocatalyst. 

Based on this premise, it is reasonable to assume that positive charge promote the establishment of attractive interactions between feed solution and membrane surface, causing rapid accumulation of foulants and, as a consequence, quicker hole-electron pair recombination. As a result, a lower resistance to fouling can be observed. Instead, negative charge assists TiO_2_ NPs with a reduced likelihood of energized holes and free electron recombination causing a longer and efficient in-situ cleaning.

### 3.6. Ionic Charge Switch to Wash Further Membranes

**Figure 9:**
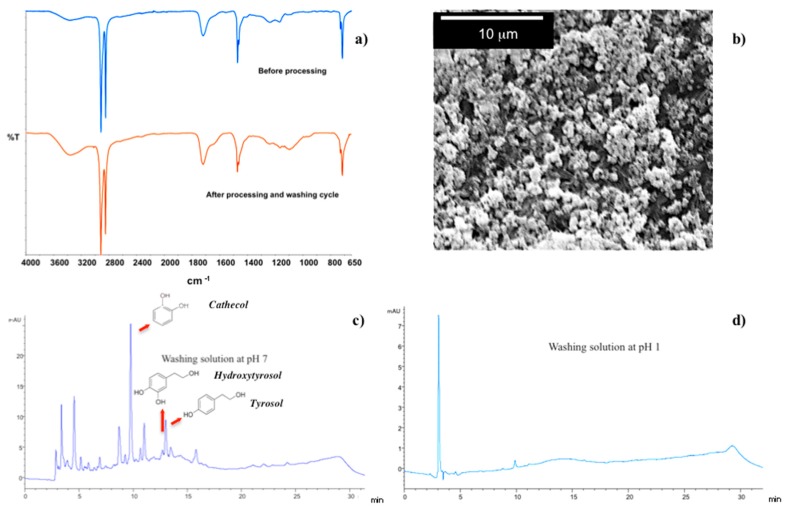
ATR spectra collected before processing and after washing the membranes at the end of microfiltration process (**a**); SEM micrograph collected onto the functional membrane surface after washing (**b**); HPLC chromatogram related to washing solutions at pH 7 (**c**) and pH 1 (**d**).

The pH responsiveness of the LBL complex takes the further advantage of removing over residues away from the membrane surface without using additional chemical reagents. At the end of the filtration process, the polymer ionic charge can be, in fact, switched causing swelling-deswelling of the hydrogel (Figure 9). 

Two successive washing cycles at pH 7 (10 min) and pH 1 (10 s) result enough to clean fully and restore the membrane surface. 

ATR spectra collected before processing and after final cleaning steps result indeed to be comparable as well as the SEM micrograph, collected onto membrane surface after washing, confirms the integrity of the LBL complex (Figure 9a,b). Also, HPLC analyses reveal the presence of removed residues, such as phenols, i.e., catechol, hydroxtyrosol and tyrosol, in washing streams at pH 7 (Figure 9b), whereas no contaminants are detectable in solution at pH 1 (Figure 9c).

Once more, the high density of negative charge at pH 7 causes the polymer chains to swell and pull out adsorbed-surface contaminants, thereby facilitating the removal of over foulants from the surface. Successively, the full protonation-taking place in acid environment has ability to get PAA segments in a shrunk state and chemically compactness, resulting in a reversible fouling and water recovery of 99.6%.

## 4. Conclusions

A dual LBL complex has been generated onto the surface of hollow fiber PE membranes in order to promote assisted self-cleaning during OMW microfiltration. A consistent containment of decline of the flux has been obtained. Polyelectrolytes, such as PAA and PDDA have been patterned with TiO_2_ NPs forming particulate-like gel structures on the PE membrane surface. Combining pH and light responsive functions, a cooperative mechanism of repulsion and assisted transfer of foulants to photocatalyst surface has been endorsed. This condition has been reached when working with OMW_4.6_ solution under irradiation, since a larger density of negative charge and a band-gap excitation has been easily induced and maintained with time. As a result, the decline of the flux has been limited over six hours of process without the need of using further chemical reagents or hard cleaning procedures, whilst the switch of ionic charge in the polyelectrolyte gel has been enough to restore the LBL complex after washing with water. The efficiency of LBL complex has been demonstrated by using PE membranes, which traditionally cause indistinctive and strong accumulation of foulants on the surface, thus resulting in a dramatic abetment of the flux. The successful result is essentially due to the formation of chemical environments, wherein assisted photodegradation can take place. 

This work is part of multitask dedicated to the design of smart membranes with enhanced fouling resistance properties. It provides new insights into the molecular study addressed at the modification of membrane surface by using combined stimuli-responsive materials. It proves how cooperative mechanisms at the stream-membrane interface can be actuated to contrast fouling events, which are envisaged as the major responsible for dramatic decline of the flux during wastewater treatment. These responsive membranes, which are designed to endorse self-cleaning events during wastewater treatment, are expected to give great propulsion to the development of competitive and eco-sustainable membrane purification processes.
